# Estimated effect of vitamin A supplementation on anaemia and anthropometric failure of Indian children

**DOI:** 10.1038/s41390-022-01969-1

**Published:** 2022-02-09

**Authors:** Rajesh Kumar Rai

**Affiliations:** 1 Society for Health and Demographic Surveillance, Suri, West Bengal India; 2 Department of Global Health and Population, Harvard T H Chan School of Public Health, Cambridge, MA USA; 3 Department of Economics, University of Goettingen, Göttingen, Germany; 4 Centre for Modern Indian Studies, University of Goettingen, Göttingen, Germany

## Abstract

**Background:**

India has an unacceptably high burden of vitamin A deficiency (VAD) among children aged 6–59 months. To mitigate VAD and its adverse effects on child health, the Indian government runs a nationwide vitamin A supplementation (VAS) programme. However, the effect of VAS in reducing child morbidity and mortality remains inconclusive and has been debated globally. In this paper, we estimate the effect of VAS on two indicators of child nutrition—anaemia (categorized into any anaemia, and mild/moderate anaemia) and anthropometric failure (categorized into stunting, wasting, and underweight) among children aged 6–59 months.

**Methods:**

Using the nationally representative 2015–2016 National Family Health Survey data set from India, we set up a quasi-experimental study design and estimated household and mother fixed-effects of VAS on select types of child anaemia and anthropometric failure.

**Results:**

Findings from both the household fixed-effects and mother fixed-effects analysis showed that VAS does not influence any types of childhood anaemia and anthropometric failure in India. We discussed the findings considering existing literature and possible limitations of the study.

**Conclusions:**

The infirm effect of Vitamin A on anaemia and anthropometric failure is probably indicative of targeted VAS intervention, as opposed to a universal VAS programme.

**Impact:**

- Effects of vitamin A supplementation (VAS) in treating child morbidity and mortality remain inconclusive, which calls for further rigorous studies.
- This study set up a quasi-experimental research design and estimated the null effect of VAS on child anaemia and childhood anthropometric failure.
- While the cautious interpretation of findings is urged, this study reliably supports targeted intervention of VAS, instead of the universal VAS programme.
- The use of nationally representative data and robust research protocol are the primary strengths of this study.

## Introduction

Vitamin A deficiency (VAD), measured as a plasma or serum retinol concentration of <0.70 μmol/l, is considered a major public health problem among children aged 6–59 months.^1^ In 2013, nearly 29% of children were estimated to have VAD in 138 low-and-middle-income countries.^2^ VAD is associated with morbidity and mortality from common childhood infections and is the world’s leading preventable cause of childhood blindness.^1^ Even mild, subclinical deficiency may increase the risk for respiratory and diarrhoeal infections among children, decrease growth rates, slow bone development, and decrease the likelihood of survival from a serious illness.^1,3–5^ Earlier studies have confirmed the pathways through which VAD could cause anaemia,^6,7^ and VAS could be an effective strategy to treat anaemia among children.^8^ The effects of VAD on anthropometric failure have also been studied,^9,10^ whereas moderate-to-severe VAD, marked by xerophthalmia, was estimated to impair normal physical growth among children.^11^ However with growing research, the effect of universal VAS in mitigating childhood mortality and morbidity has been questioned.

To combat VAD, the World Health Organization (WHO) devised a guideline^1^ of vitamin A supplementation (VAS) for the population where the prevalence of night blindness is 1% or higher among children 24–59 months of age or where the prevalence of VAD is 20% or higher among infants and children aged 6–59 months. WHO recommended that infants aged 6–11 months should receive 100,000 IU or international units (30 mg RE or milligram retinol equivalent) of vitamin A once and children aged 12–59 months should receive 200,000 IU (60 mg RE) of vitamin A every 4–6 months. However, this recommendation of universal periodic VAS has been debated and challenged,^12,13^ and the most recent evidence questions the effect of VAS in reducing child deaths, whereas most deaths from measles and diarrhoea appeared to be preventable in the present time.^12,14^ Thus the effects of universal VAS programme on child health remains inconclusive, which demands further evidence to understand the need for universal periodic VAS in improving child health.

India’s population suffers from a high burden of micronutrient deficiency, with an estimated VAD of 19% (95% confidence interval: 9–29%).^15^ With a population of over 124 million children aged 6–59 months, India also runs a nationwide public VAS programme.^16^ India adopted the VAS guideline developed by WHO and a total of nine oral doses of VAS was recommended for all children by their fifth birthday.^16^ The Integrated Child Development Services (ICDS) programme established under the Department of Women and Child Development was tasked with the periodic distribution of VAS doses to children to prevent VAD.^16^ However, in line with the ongoing debate about the need and effectiveness of VAS programmes globally, this debate has also grown in India.^17–20^ A recent study^17^ used the Comprehensive National Nutrition Survey (CNNS) data of children aged 1–5 years and concluded that the national prevalence of VAD is 15.7% and a targeted approach (as opposed to universal) of VAS intervention was recommended considering India’s progress in reduction of infant and child mortality, immunization coverage, and recent initiation of oil and milk fortification with vitamin A. However, these conclusions implied reasonable doubts about existing research findings and a careful interpretation of findings was urged.^12^ Responding to the CNNS based study,^17^ a group of researchers welcomed the use of improved targeting or prioritization of VAS delivery to population groups at higher risk of VAD,^21^ whereas a letter to the Editor^22^ pointed out the insufficiency of evidence for proposing targeting in VAS strategy. The effectiveness of VAS was also questioned by the Deworming and Enhanced Vitamin A supplementation study, a cluster-randomized trial, the largest program evaluation of universal VAS, conducted in the state of Uttar Pradesh in India, which found no benefits on all-cause or cause-specific mortality.^23^

Therefore, while the need for universal periodic VAS intervention among children in India has been questioned, it is worth exploring if there is any estimated impact of VAS on child health using the latest publicly available nationally representative data. Using appropriate MeSH (Medical Subject Headings) terms, we searched for the existing literature on this issue in India and found no recent national study has to date tried to assess the effect of VAS on various child health indicators in India. To fill this evidence gap, using a nationally representative data set from India, we set up a quasi-experimental fixed-effects (household-and-mother-fixed-effects) study design to estimate the effect of VAS on childhood anaemia (categorized into any anaemia, and mild/moderate anaemia), and anthropometric failure (categorized into stunting, wasting, and underweight), among children aged 6–59 months in India. In the context of the availability of its first national estimates of VAD among children aged 1–5 years,^23,24^ this study will be a timely effort and could be a significant research contribution for discussion about the need for universal periodic VAS programmes to correct childhood anaemia and anthropometric failure in India.

## Methods

### Data set

The data set used for this study was retrieved from the fourth wave of nationally representative cross-sectional standard Demographic and Health Survey of India, commonly known as the 2015–2016 National Family Health Survey (or NFHS-4).^25^ Financially supported by the Ministry of Health and Family Welfare, NFHS-4 provides information on population, health, and nutrition for 37 state/union territories of India, credibly used by programmes and policymakers to guide national public health policy. NFHS-4 used the 2011 Census of India sampling frame and adopted a two-stage stratified sampling design to select the primary sampling unit (PSU) in rural (villages) and urban areas (Census Enumeration Blocks (CEB)). Within each rural stratum, villages were selected from the sampling frame with probability proportional to size (PPS). In each stratum, six approximately equal substrata were created by crossing three substrata, each created based on the estimated number of households in each village, with two substrata, each created based on the percentage of the population belonging to scheduled castes (SCs) and scheduled tribes (STs). CEBs in urban areas were sorted according to the percentage of the SC/ST population in each CEB, and sample CEBs were selected with PPS sampling. With an over 97% household response rate, a total of 601,509 households were selected to interview 699,686 women and 112,122 men in NFHS-4. More about the sampling procedure can be obtained from its published report.^25^

We used the children recode file of NFHS-4 having information for 259,627 children. On behalf of children born in the 5 years preceding the survey date, mothers responded and helped NFHS-4 gather information on child mortality, child nutrition, childhood diseases and several other child health indicators. It is worth mentioning that unlike the earlier three rounds of NFHS, NFHS-4 covers information on a large number of children helped in setting up this quasi-experimental study. To select the final sample eligible for the household-and-mother-fixed-effects analysis, a four-stage sample recruiting method was followed. At stage I, all outcome variables and control variables (VAS and other variables) were constructed. In stage II, all the observations of children aged <6 months and >59 months of age were dropped from the data set, keeping children aged 6–59 months for the analysis. Stage III involved the computation of unique identification numbers for all households and all mothers, and selected households that had at least two children for the analysis of household fixed-effects, and for mother fixed-effects, we developed a data set with mothers who had at least two children. Finally, stage IV included preparation of the data set that included only households or mothers whose children had differing status in receiving VAS. Once the data set is finalized, a total number of 21,475 and 21,021 children were found to be eligible for running household fixed-effects analysis for anaemia and anthropometric failures, respectively. On the other hand, for analysing mother fixed-effects, a total of 16,676, and 16,298 children were included to analyse anaemia and anthropometric failures, respectively.

### Outcome events

A total of five outcome events were analysed. They are anaemia (categorized into any anaemia and mild/moderate anaemia) and anthropometric failure (categorized into stunting, wasting, and underweight). Indian children experience a high prevalence of anaemia (58.6% as in 2015–2016) and little improvement has been recorded in the past decade.^26^ WHO guidelines^27^ classify a haemoglobin (Hb) level of <11 g/dl as having any anaemia, whereas Hb of 7–10.9 g/dl is defined as mild/moderate anaemia. NFHS-4 provides Hb estimates of children adjusted for the altitude of their residence. Using HemoCue Hb 201+ analyser, NFHS-4 measured Hb level from the capillary blood sample of children aged 6–59 months. After excluding biologically implausible values, anthropometric failure (stunting, wasting, and underweight) were computed. Stunting was defined as <−2 standard deviations from median height for age of the reference population; wasting was defined as <−2 standard deviations from median weight for height of the reference population, and underweight was defined as <−2 standard deviations from median weight for age of the reference population.^28^ For child anthropometry, “SECA 874 U digital scale” was used for weighing, “SECA 213 Stadiometer” for measuring height, and “SECA 417 Infantometer” was used for measuring the length of children aged <2 years or <85 cm. NFHS-4 reported that 38.4, 21, and 35.8% of children under-five were stunted, wasted, and underweight, respectively.^25^

### VAS and other control variables

As a primary control variable, the information on VAS was used. In NFHS-4, women were asked about each living child born in 2011 or later—“within the last 6 months, was (the child) given a vitamin A dose?” A sample of vitamin A dose was shown to respondents to minimize recall errors. As stated earlier, children aged between 6 months and 59 months are expected to receive nine doses of VAS in total.^1^ Although NFHS-4 does not include the number of VAS doses a child received, the information on receipt of VAS in the 6 months preceding the survey date indicates if the receipt of VAS is timely and one may expect that child receiving timely VAS will have better health than children who did not receive any VAS 6 months preceding the survey date. As estimated from NFHS-4 data, nearly 58.6% of children aged 6–59 months received VAS dose, which is a threefold increase in VAS from NFHS 2005–2006.^25^

Apart from the primary variable of interest, a set of variables representing child characteristics were used. They are—if the child belonged to women who had twin/multiple births, age of the children, sex, birth order, and if the children received any benefit from *Anganwadi* centres (AWCs; meaning “courtyard shelter”). In NFHS-4, mothers were asked—“during the last 12 months, has (your child) received any benefits from the *Anganwadi* centre?” Any benefits included supplementary food, growth monitoring, immunizations, health check-ups, or education. The Indian government established AWCs in 1975 as part of the ICDS programme to combat hunger and malnutrition among women and children. India currently has over 1.3 million operational AWC managed by an *anganwadi* worker.^29^ Aside from other responsibilities of *Anganwadi* workers for children, the 2013 National Food Security Act mandated take-home ration or morning snacks and hot cooked meals to children aged 6 months to 6 years at AWCs.^30^ This information on services from AWCs is useful for this study to understand whether exposure to such social security programmes could help strengthen childhood nutrition.

Guided by existing literature,^31–36^ a set of variables representing maternal characteristics was also constructed. They are mother’s age at first birth, mother’s education, maternal body mass index (BMI), tobacco and alcohol use of mothers, if the mother was pregnant or breastfeeding at the time of the survey, if the mother lives with husband/partner, and diabetes and hypertension status of mothers. Maternal BMI for the Indian population (weight in kilograms divided by the square of height in metres) was constructed as per WHO guidelines.^37^ Individuals with blood glucose levels of ≥141 mg/dl or those on medication for diabetes were defined as diabetic.^25^ Individuals were defined as hypertensive if their systolic blood pressure level reading was ≥140 mm of mercury or mmHg, or diastolic blood pressure reading was ≥90 mmHg, or if they were on medication to mitigate hypertension.^25^

The variable presenting three doses of diphtheria, pertussis, and tetanus (DPT3) immunization was also constructed to control for variation in receipt of vaccination over time, despite most of the unobserved heterogeneity removed by using household and mother fixed-effects,^38,39^ while assessing the effect of VAS. It is worth mentioning that the inclusion of additional control variables helped control for intrahousehold variability of child health as one household may house more than one mother.

### Statistical approach

To assess the effect of VAS on childhood anaemia and anthropometric failure, this study opted for conditional logistic regression, adjusted for household or mother fixed-effects. Fixed-effects design, a widely used quasi-experimental study design, is the generalization of difference-in-difference designs, and individuals can be measured under different treatment statuses but are nested within a larger level, allowing for control of all observed and unobserved factors common among all individuals belonging to the same entity (here it is the same mother or the same household).^40^ For example: in cross-sectional analysis, controlling for all factors that are shared by siblings, such as having the same household/family^38^ or the same mother.^38,39^ For this study, the conditional logistic (household fixed-effects) regression was specified as below:

$$Y_{ih} = \beta _0 + \beta _1V_{ih} + \beta _2X_{ih}^{{{{{{\rm{child}}}}}}} + \beta _3X_{ih}^{{{{{{\rm{mother}}}}}}} + \delta _h + \varepsilon _{ih}$$

$Y_{ih}$ is the select outcome event of child $i$ in household $h$. $V_{ih}$ is child $i$’s receipt of VAS, and $\beta _1$ is the main parameter of interest: the association between receipt of VAS child health outcome. $X_{ih}^{{{{{{\rm{child}}}}}}}$ is a vector containing child-specific control variables including DPT vaccination status, $X_{ih}^{{{{{{\rm{mother}}}}}}}$ is a vector containing mother-specific control variable, $\delta _h$ is the household fixed-effects, and $\varepsilon _{ih}$ is the error term. In mother fixed-effects regressions, the vector of mother-specific control variables is dropped and $\delta _h$ counts as the mother fixed-effects, whereas $h$ represents the mother instead of the household for all variables in the regression models.

To run the above-specified regression, we computed all the outcome events as binary terms—“1” in case of anaemia, mild/moderate anaemia, stunting, wasting, and underweight, otherwise coded as “0”. For each outcome event, we ran two models—model I included the variables representing children and/or mother characteristics without controlling the effect of DPT3, whereas model II controlled the effect of DPT3, and we repeated this analysis with household fixed-effects and mother fixed-effects. It is worth mentioning that while running the regression models, either for household or mother fixed-effects, the observation was dropped if the model did not find enough variation for outcome events for the same household or same mother. For example: running the household fixed-effects for anaemia included 21,475 observations, but 11,453 samples were excluded automatically from the regression model. This process may lead to a biased estimate.^41^ However, using conditional logistic regression instead of unconditional logistic regression helped minimize the bias, and the estimates are deemed robust.^42^ Prior to running conditional logistic regression models, we examined the distribution of the sample in the analytical model to understand the presence of sample selection bias. Except for counts, appropriate sample weighting was used to run the analysis. The statistical software Stata version 14^43^ was used to execute the analysis, and “svy” suite available with Stata was applied for all estimations, rendering robust estimates with reliable standard error.

## Results

Table 1 displays the descriptive statistics of the sample included for household fixed-effects. Nearly half of the sample selected for anaemia and anthropometric failure had received VAS. A nearly equal proportion of male or female participants were included. Most mothers had their first child between the age of 18 and 24 years. Nearly 29% of mothers were underweight (BMI of <18.5 kg/m^2^). Nearly 6 and 1% of mothers were tobacco and alcohol users, respectively. Nearly 3% of mothers were diabetic, and 1% of all mothers included were hypertensive. Over 77% of all children received all three doses of DPT vaccinations.

**Table 1:** Sample distribution of child health indicators by receipt of vitamin A supplementation (VAS) and background characteristics of mothers and their children.

	Anaemia, Prop. (SD)	Anthropometric failure, Prop. (SD)
Received VAS		
No	0.50 (0.002)	0.50 (0.002)
Yes	0.50 (0.002)	0.50 (0.002)
Twin/multiple birth		
No	0.99 (0.001)	0.99 (0.001)
Yes	0.01 (0.001)	0.01 (0.001)
Age (years)		
0	0.15 (0.003)	0.16 (0.003)
1	0.23 (0.004)	0.23 (0.004)
2	0.19 (0.003)	0.19 (0.003)
3	0.21 (0.003)	0.21 (0.003)
4	0.22 (0.003)	0.21 (0.003)
Sex		
Male	0.50 (0.005)	0.49 (0.005)
Female	0.50 (0.005)	0.51 (0.005)
Birth order		
1	0.32 (0.004)	0.33 (0.004)
2	0.36 (0.003)	0.37 (0.003)
3	0.17 (0.003)	0.17 (0.003)
≥4	0.14 (0.004)	0.14 (0.004)
Received benefits from *Anganwadi* centre		
No	0.41 (0.006)	0.41 (0.006)
Yes	0.59 (0.006)	0.59 (0.006)
Mother’s age at first birth (years)		
<18	0.14 (0.005)	0.14 (0.005)
18–24	0.75 (0.006)	0.75 (0.006)
25–30	0.10 (0.004)	0.10 (0.004)
≥31	0.01 (0.002)	0.01 (0.002)
Maternal education		
No education	0.33 (0.006)	0.32 (0.006)
Primary	0.16 (0.005)	0.16 (0.005)
Secondary or higher	0.51 (0.007)	0.52 (0.007)
Maternal BMI (kg/m^2^)		
<18.5	0.29 (0.006)	0.29 (0.006)
18.5–22.9	0.50 (0.007)	0.49 (0.007)
≥23.0	0.22 (0.006)	0.22 (0.006)
Maternal tobacco use		
Non-user	0.94 (0.003)	0.94 (0.003)
User	0.06 (0.003)	0.06 (0.003)
Maternal alcohol use		
Non-user	0.99 (0.001)	0.99 (0.001)
User	0.01 (0.001)	0.01 (0.001)
Currently pregnant		
No/unsure	0.92 (0.003)	0.92 (0.003)
Yes	0.08 (0.003)	0.08 (0.003)
Currently breastfeeding		
No	0.23 (0.006)	0.23 (0.006)
Yes	0.77 (0.006)	0.77 (0.006)
Has husband/partner		
No	0.01 (0.001)	0.01 (0.001)
Yes	0.99 (0.001)	0.99 (0.001)
Maternal diabetes status		
Non-diabetic	0.97 (0.002)	0.97 (0.002)
Diabetic	0.03 (0.002)	0.03 (0.002)
Maternal hypertension status		
Non-hypertensive	0.99 (0.001)	0.99 (0.001)
Hypertensive	0.01 (0.001)	0.01 (0.001)
Received three doses of DPT vaccinations		
No	0.23 (0.005)	0.23 (0.005)
Yes	0.77 (0.005)	0.77 (0.005)
*n*	21,475	21,021

The proportion may not add to 1 due to rounding.

*BMI* body mass index, *DPT* diphtheria, pertussis, and tetanus, *Prop.* proportion, *SD* standard deviation.

Tables 2 and 3 represent the findings from household fixed-effects showing the effect of VAS and other control variables on anaemia (any anaemia and mild/moderate anaemia), and anthropometric failure (stunting, wasting, and underweight, respectively. Both for model I (without adjustment of DPT3) and model II (with adjustment of DPT3), the result from household fixed-effects showed that VAS has no effect on anaemia or anthropometric failure. Tables S1 and S2 (online Supplementary Tables) tested the effects of mother fixed-effects on anaemia (any anaemia and mild/moderate anaemia), and anthropometric failure (stunting, wasting, and underweight), respectively. Like household fixed-effects, mother fixed-effects did not show any effects of VAS on childhood anaemia and anthropometric failure. Online supplementary Tables S3 and S4 furnish the distribution of the analytical sample included in household fixed-effects by the status of childhood anaemia and anthropometric failure, and sample distribution appeared reasonably comparable, thus reducing the probability of sample selection bias.

**Table 2:** Household fixed-effects regression of anaemia (any anaemia, and moderate/mild anaemia) on receipt of vitamin A supplementation (VAS) and control variables.

	Any anaemia				Moderate/mild anaemia			
	Model I OR (95% CI)	*p*	Model II OR (95% CI)	*p*	Model I OR (95% CI)	*p*	Model II OR (95% CI)	*p*
Received VAS								
No	1.00		1.00		1.00		1.00	
Yes	1.02 (0.91–1.13)	0.782	1.01 (0.91–1.13)	0.803	1.01 (0.91–1.11)	0.900	1.01 (0.91–1.11)	0.897
Twin/multiple births								
No	1.00		1.00		1.00		1.00	
Yes	1.22 (0.64–2.33)	0.539	1.22 (0.64–2.34)	0.541	0.95 (0.51–1.77)	0.875	0.95 (0.51–1.77)	0.876
Age (years)								
0	1.00		1.00		1.00		1.00	
1	1.13 (0.91–1.41)	0.276	1.13 (0.91–1.40)	0.285	1.04 (0.84–1.28)	0.722	1.04 (0.84–1.28)	0.720
2	0.73 (0.60–0.89)	0.002	0.73 (0.60–0.88)	0.001	0.72 (0.60–0.87)	0.001	0.72 (0.60–0.87)	0.001
3	0.43 (0.35–0.53)	<0.001	0.43 (0.35–0.53)	0.001	0.46 (0.38–0.56)	<0.001	0.46 (0.38–0.56)	<0.001
4	0.32 (0.26–0.40)	<0.001	0.32 (0.26–0.40)	<0.001	0.35 (0.28–0.43)	<0.001	0.35 (0.28–0.43)	<0.001
Sex								
Male	1.00		1.00		1.00		1.00	
Female	0.92 (0.80–1.05)	0.228	0.92 (0.80–1.05)	0.227	0.94 (0.82–1.07)	0.332	0.94 (0.82–1.07)	0.332
Birth order								
1	1.00		1.00		1.00		1.00	
2	1.06 (0.92–1.23)	0.411	1.06 (0.92–1.23)	0.413	1.07 (0.93–1.23)	0.351	1.07 (0.93–1.23)	0.350
3	1.12 (0.90–1.39)	0.299	1.12 (0.90–1.39)	0.301	1.14 (0.92–1.40)	0.233	1.14 (0.92–1.40)	0.233
≥4	1.19 (0.88–1.61)	0.268	1.19 (0.88–1.60)	0.270	1.14 (0.85–1.52)	0.378	1.14 (0.85–1.52)	0.377
Received benefits from *Anganwadi* centre								
No	1.00		1.00		1.00		1.00	
Yes	0.98 (0.82–1.16)	0.793	0.98 (0.82–1.16)	0.792	0.98 (0.83–1.16)	0.839	0.98 (0.83–1.16)	0.839
Mother’s age at first birth (years)								
<18	1.00		1.00		1.00		1.00	
18–24	0.86 (0.60–1.23)	0.397	0.86 (0.60–1.22)	0.395	0.89 (0.63–1.25)	0.508	0.89 (0.63–1.26)	0.508
25–30	0.83 (0.51–1.34)	0.444	0.83 (0.51–1.34)	0.443	0.87 (0.55–1.39)	0.570	0.87 (0.55–1.39)	0.570
≥31	0.80 (0.23–2.77)	0.729	0.80 (0.23–2.75)	0.725	0.67 (0.19–2.31)	0.521	0.67 (0.19–2.31)	0.522
Maternal education								
No education	1.00		1.00		1.00		1.00	
Primary	0.73 (0.50–1.08)	0.117	0.73 (0.50–1.08)	0.117	0.77 (0.53–1.11)	0.163	0.77 (0.53–1.11)	0.163
Secondary or higher	0.83 (0.60–1.14)	0.255	0.83 (0.60–1.14)	0.255	0.84 (0.62–1.15)	0.276	0.84 (0.62–1.15)	0.276
Maternal BMI (kg/m^2^)								
<18.5	1.00		1.00		1.00		1.00	
18.5–22.9	1.10 (0.84–1.43)	0.488	1.10 (0.84–1.43)	0.493	1.05 (0.82–1.36)	0.688	1.05 (0.82–1.36)	0.687
≥23.0	1.20 (0.82–1.76)	0.354	1.20 (0.82–1.75)	0.357	1.12 (0.78–1.61)	0.548	1.12 (0.78–1.61)	0.547
Maternal tobacco use								
Non-user	1.00		1.00		1.00		1.00	
User	1.28 (0.65–2.49)	0.475	1.28 (0.65–2.49)	0.472	1.34 (0.70–2.58)	0.382	1.34 (0.70–2.58)	0.382
Maternal alcohol use								
Non-user	1.00		1.00		1.00		1.00	
User	1.74 (0.20–14.79)	0.614	1.74 (0.20–14.83)	0.614	1.70 (0.21–13.61)	0.615	1.70 (0.21–13.60)	0.615
Currently pregnant								
No/unsure	1.00		1.00		1.00		1.00	
Yes	1.41 (1.02–1.95)	0.039	1.41 (1.02–1.95)	0.039	1.53 (1.12–2.10)	0.008	1.53 (1.12–2.10)	0.008
Currently breastfeeding								
No	1.00		1.00		1.00		1.00	
Yes	1.21 (0.93–1.57)	0.163	1.20 (0.93–1.57)	0.166	1.26 (0.98–1.62)	0.076	1.26 (0.98–1.63)	0.076
Has husband/partner								
No	1.00		1.00		1.00		1.00	
Yes	1.33 (0.62–2.89)	0.465	1.33 (0.61–2.88)	0.470	1.28 (0.60–2.72)	0.524	1.28 (0.60–2.72)	0.523
Maternal diabetes status								
Non-diabetic	1.00		1.00		1.00		1.00	
Diabetic	1.13 (0.57–2.25)	0.731	1.13 (0.57–2.25)	0.730	1.04 (0.53–2.07)	0.903	1.04 (0.53–2.07)	0.903
Maternal hypertension status								
Non-hypertensive	1.00		1.00		1.00		1.00	
Hypertensive	1.81 (0.62–5.28)	0.276	1.81 (0.62–5.28)	0.275	1.89 (0.67–5.38)	0.231	1.89 (0.67–5.38)	0.231
Received three doses of DPT vaccinations	nm				nm			
No			1.00				1.00	
Yes			1.03 (0.85–1.24)	0.793			0.99 (0.83–1.20)	0.954
*n* (included in the model)	10,022		10,022		10,464		10,464	
*n* (dropped from the model)	11,453		11,453		11,011		11,011	

nm: not included in the model; *p*: level of significance.

*BMI* body mass index, *CI* confidence interval, *DPT* diphtheria, pertussis, and tetanus, *OR* odds ratio.

**Table 3:** Household fixed-effects regression of anthropometric failure (stunting, wasting, and underweight) on receipt of vitamin A supplementation (VAS) and control variables.

	Stunting				Wasting				Underweight			
	Model I OR (95% CI)	*p*	Model II OR (95% CI)	*p*	Model I OR (95% CI)	*p*	Model II OR (95% CI)	*p*	Model I OR (95% CI)	*p*	Model II OR (95% CI)	*p*
Received VAS												
No	1.00		1.00		1.00		1.00		1.00		1.00	
Yes	1.00 (0.91–1.10)	0.957	1.00 (0.91–1.10)	0.959	0.96 (0.86–1.08)	0.501	0.97 (0.86–1.08)	0.560	1.01 (0.92–1.11)	0.885	1.02 (0.92–1.12)	0.729
Twin/multiple births												
No	1.00		1.00		1.00		1.00		1.00		1.00	
Yes	1.21 (0.51–2.84)	0.667	1.21 (0.51–2.86)	0.659	2.23 (0.82–6.08)	0.117	2.27 (0.84–6.12)	0.104	1.11 (0.55–2.21)	0.776	1.12 (0.56–2.22)	0.747
Age (years)												
0	1.00		1.00		1.00		1.00		1.00		1.00	
1	3.60 (2.92–4.44)	<0.001	3.64 (2.95–4.49)	<0.001	0.68 (0.55–0.84)	<0.001	0.69 (0.55–0.85)	0.001	1.61 (1.31–1.99)	<0.001	1.65 (1.34–2.03)	<0.001
2	4.33 (3.49–5.36)	<0.001	4.37 (3.51–5.44)	<0.001	0.40 (0.32–0.51)	<0.001	0.41 (0.32–0.52)	<0.001	1.69 (1.38–2.07)	<0.001	1.73 (1.41–2.13)	<0.001
3	4.29 (3.46–5.33)	<0.001	4.33 (3.49–5.37)	<0.001	0.42 (0.33–0.53)	<0.001	0.43 (0.34–0.54)	<0.001	1.86 (1.50–2.290)	<0.001	1.90 (1.54–2.350)	<0.001
4	3.04 (2.42–3.81)	<0.001	3.06 (2.43–3.84)	<0.001	0.36 (0.28–0.47)	<0.001	0.37 (0.28–0.47)	<0.001	1.68 (1.34–2.10)	<0.001	1.71 (1.36–2.14)	<0.001
Sex												
Male	1.00		1.00		1.00		1.00		1.00		1.00	
Female	1.00 (0.89–1.14)	0.939	1.00 (0.89–1.13)	0.962	0.87 (0.75–1.01)	0.065	0.87 (0.75–1.010)	0.065	0.99 (0.88–1.12)	0.867	0.98 (0.87–1.11)	0.802
Birth order												
1	1.00		1.00		1.00		1.00		1.00		1.00	
2	1.32 (1.14–1.52)	<0.001	1.31 (1.14–1.52)	<0.001	1.01 (0.86–1.20)	0.895	1.01 (0.85–1.20)	0.898	1.12 (0.97–1.29)	0.125	1.12 (0.97–1.29)	0.133
3	1.54 (1.23–1.93)	<0.001	1.54 (1.23–1.92)	<0.001	1.16 (0.89–1.50)	0.266	1.16 (0.89–1.50)	0.272	1.43 (1.14–1.80)	0.002	1.43 (1.14–1.79)	0.002
≥4	2.42 (1.76–3.34)	<0.001	2.42 (1.75–3.34)	<0.001	1.11 (0.77–1.60)	0.570	1.11 (0.77–1.59)	0.587	2.05 (1.50–2.82)	<0.001	2.05 (1.49–2.81)	<0.001
Received benefits from *Anganwadi* centre												
No	1.00		1.00		1.00		1.00		1.00		1.00	
Yes	0.85 (0.71–1.02)	0.074	0.85 (0.71–1.02)	0.078	1.10 (0.91–1.34)	0.335	1.11 (0.91–1.35)	0.317	0.98 (0.83–1.16)	0.802	0.99 (0.83–1.17)	0.892
Mother’s age at first birth (years)												
<18	1.00		1.00		1.00		1.00		1.00		1.00	
18–24	1.09 (0.76–1.57)	0.645	1.08 (0.75–1.56)	0.662	1.70 (1.01–2.85)	0.044	1.71 (1.03–2.85)	0.040	1.33 (0.91–1.94)	0.135	1.33 (0.92–1.94)	0.132
25–30	1.38 (0.78–2.45)	0.273	1.37 (0.77–2.45)	0.280	2.48 (1.23–5.00)	0.011	2.52 (1.26–5.05)	0.009	2.31 (1.23–4.34)	0.009	2.29 (1.22–4.32)	0.010
≥31	1.65 (0.55–4.98)	0.375	1.64 (0.54–4.94)	0.381	0.94 (0.25–3.57)	0.926	0.95 (0.25–3.58)	0.935	1.14 (0.31–4.26)	0.842	1.12 (0.30–4.16)	0.870
Maternal education												
No education	1.00		1.00		1.00		1.00		1.00		1.00	
Primary	0.87 (0.57–1.32)	0.507	0.87 (0.57–1.33)	0.525	1.25 (0.75–2.07)	0.396	1.24 (0.75–2.07)	0.398	0.99 (0.63–1.57)	0.976	1.01 (0.64–1.58)	0.977
Secondary or higher	1.07 (0.75–1.53)	0.711	1.07 (0.75–1.53)	0.710	1.15 (0.73–1.81)	0.536	1.15 (0.73–1.81)	0.542	1.02 (0.70–1.48)	0.925	1.02 (0.70–1.49)	0.921
Maternal BMI (kg/m^2^)												
<18.5	1.00		1.00		1.00		1.00		1.00		1.00	
18.5–22.9	1.03 (0.78–1.36)	0.818	1.04 (0.78–1.37)	0.806	0.72 (0.51–1.02)	0.063	0.72 (0.51–1.02)	0.063	0.85 (0.63–1.13)	0.259	0.85 (0.63–1.13)	0.262
≥23.0	0.64 (0.43–0.96)	0.032	0.65 (0.43–0.96)	0.032	0.85 (0.54–1.33)	0.469	0.84 (0.53–1.33)	0.463	0.74 (0.48–1.14)	0.178	0.75 (0.49–1.15)	0.182
Maternal tobacco use												
Non-user	1.00		1.00		1.00		1.00		1.00		1.00	
User	1.12 (0.64–1.95)	0.692	1.11 (0.63–1.93)	0.722	1.12 (0.49–2.55)	0.792	1.11 (0.48–2.53)	0.812	0.92 (0.44–1.95)	0.837	0.93 (0.44–1.93)	0.836
Maternal alcohol use												
Non-user	1.00		1.00		1.00		1.00		1.00		1.00	
User	0.16 (0.03–0.92)	0.040	0.16 (0.03–0.92)	0.040	1.67 (0.16–17.60)	0.671	1.62 (0.15–17.13)	0.688	0.67 (0.15–2.97)	0.601	0.65 (0.14–2.93)	0.573
Currently pregnant												
No/unsure	1.00		1.00		1.00		1.00		1.00		1.00	
Yes	1.06 (0.71–1.59)	0.777	1.06 (0.70–1.59)	0.784	1.46 (0.95–2.25)	0.085	1.45 (0.94–2.24)	0.090	0.96 (0.66–1.40)	0.849	0.96 (0.66–1.40)	0.836
Currently breastfeeding												
No	1.00		1.00		1.00		1.00		1.00		1.00	
Yes	1.33 (1.00–1.76)	0.046	1.33 (1.01–1.76)	0.046	1.04 (0.75–1.43)	0.818	1.04 (0.75–1.43)	0.829	1.40 (1.05–1.86)	0.022	1.40 (1.05–1.86)	0.021
Has husband/partner												
No	1.00		1.00		1.00		1.00		1.00		1.00	
Yes	1.18 (0.46–3.06)	0.729	1.19 (0.46–3.08)	0.723	2.94 (0.75–11.59)	0.122	2.96 (0.76–11.58)	0.119	1.47 (0.73–2.96)	0.283	1.50 (0.74–3.02)	0.257
Maternal diabetes status												
Non-diabetic	1.00		1.00		1.00		1.00		1.00		1.00	
Diabetic	1.47 (0.64–3.38)	0.367	1.47 (0.64–3.39)	0.361	1.30 (0.58–2.92)	0.524	1.30 (0.58–2.94)	0.522	1.18 (0.47–2.96)	0.724	1.18 (0.47–2.96)	0.717
Maternal hypertension status												
Non-hypertensive	1.00		1.00		1.00		1.00		1.00		1.00	
Hypertensive	0.68 (0.25–1.82)	0.443	0.69 (0.26–1.84)	0.461	2.21 (0.73–6.72)	0.160	2.22 (0.73–6.71)	0.159	1.04 (0.37–2.87)	0.946	1.04 (0.38–2.87)	0.936
Received three doses of DPT vaccinations	nm				nm				nm			
No			1.00				1.00				1.00	
Yes			0.93 (0.77–1.13)	0.466			0.90 (0.72–1.13)	0.373			0.82 (0.68–0.99)	0.038
*n* (included in the model)	9393		9393		6187		6187		8500		8500	
*n* (dropped from the model)	11,628		11,628		14834		14834		12,521		12,521	

nm: not included in the model; *p*: level of significance.

*BMI* body mass index, *CI* confidence interval, *DPT* diphtheria, pertussis, and tetanus, *OR* odds ratio.

## Discussion

India, with a population of over 1.3 billion, has a high burden of micronutrient deficiencies among children, and VAD is one among them.^15,44^ To mitigate VAD, India adopted the WHO guideline for VAS^1^ and recommends a universal periodic dose of vitamin A for children aged 6–59 months to prevent child mortality and morbidity including infections.^16^ However, researchers, donors, and policymakers have questioned the VAS programme and recommended a targeted approach to VAS may be introduced instead of universal VAS.^17–20^ In the wake of this debate of universal versus targeted approaches to VAS, using nationally representative NFHS-4 data,^25^ this study took on a quasi-experimental (household and mother) fixed-effects study design to assess the effect of VAS on anaemia and anthropometric failure among children 6–59 months.

Findings from both household and mother fixed-effects revealed that VAS has no effect on anaemia (categorized into any anaemia and mild/moderate anaemia) and anthropometric failure (categorized into stunting, wasting, and underweight). These findings resonate with the recent body of evidence including systematic reviews. A systematic review^45^ suggested that positive effects of VAS appear limited to populations with acute and chronic undernutrition, and India suffers from high burdens of growth failure,^46^ anaemia,^26^ and micronutrient deficiencies.^24^ Another systematic review which included 16 studies from India suggested that VAS has no effect on the incidence of respiratory disease or hospitalizations due to diarrhoea or pneumonia.^3^ A recent meta-analysis of Indian studies also concluded that children aged 6–59 months who received VAS had no survival benefits.^47^ All these studies support the findings of this study that universal periodic doses of VAS may not be useful in preventing anaemia, anthropometric failure, and childhood infections in India.

The findings of this study should be interpreted considering its limitations. First, this study analysed VAS among children aged 6–59 months, whereas the Government of India encourages VAS for children aged 9-59 months.^16^ The purpose of analysing the age group 6–59 months was to have a global perspective as per the guideline developed by WHO. However, we ran the analysis for children aged 9–59 months (data not shown separately) and the findings did not differ from the analysis for children aged 6–59 months. Second, for grouping the anaemia level, we could not consider severe anaemia (Hb <7 g/dl) as a potential outcome measure because of the reasonably low sample size to run the analysis. Third, similar reasoning applies for excluding childhood diarrhoea, and acute respiratory infections (ARI) from the analysis. Fourth, our fixed-effects model relies on variation in VAS status among siblings and includes only observations from households, or mothers, with more than one child, excluding children with families with one child. This process reduces the sample size, and it may affect the representativeness of the sample. However, this should not raise concerns about the findings because apart from controlling for observed and unobserved heterogeneity, the regression models also controlled for underlying maternal factors which could affect the outcome of interest.^37^ Fifth, due to the unavailability of information on VAS status of children reported dead during the survey, this study could not estimate the effect of VAS on child mortality. Sixth, although it is not the objective of the study, for an execution point of view of VAS programme in India, the future study may explore the possible reasons on why, within a household or child belonged to the same mother, one child received VAS while another did not. Seventh, most information collected on children is based on mother’s recall, thus one cannot completely rule out the possibility of recall errors or social desirability bias. Finally, the findings of this study must be treated as the estimated effect, which has the potential of undermining the true effect of VAS on child nutrition. Simply put, the absence of estimated effect of VAS on child health indicators are not always indicative of true effects.

Despite these limitations, this study is the first of its kind to use nationally representative data to estimate that universal periodic VAS may not be an effective strategy to mitigate select childhood nutrition and diseases, which could be suggestive of targeted VAS intervention. If universal VAS is not helping to improve childhood nutrition and diseases, the targeted VAS programme might not only be a cost-effective strategy but will also be less of a burden on India’s public health system.

## Supplementary information

Supplementary Material

## Data Availability

The 2015–2016 National Family Health Survey data set used for this study is available at https://dhsprogram.com/.
