# Supplementary material for: Estimated effect of vitamin A supplementation on anaemia and anthropometric failure of Indian children
Source: Pediatr Res. 2022 Feb 9;91(5):1263–71. doi: 10.1038/s41390-022-01969-1 (PMC9122827; doi:10.1038/s41390-022-01969-1)
Supplement: Supplementary file 1 — Supplementary Material [file 41390_2022_1969_MOESM1_ESM.pdf]

Estimated effect of vitamin A supplementation on anaemia and anthropometric failure of Indian children

Rajesh Kumar Rai

Online supplementary material

Table S1. Mother fixed-effects regression of anaemia (any anaemia, and moderate/mild anaemia) on receipt of vitamin A supplementation (VAS) and control variables.

	Any anaemia				Moderate/mild anaemia			
	Model I OR (95% CI)	p	Model II OR (95% CI)	p	Model I OR (95% CI)	p	Model II OR (95% CI)	p
**Received VAS**								
No	1.00		1.00		1.00		1.00	
Yes	1.04 (0.92-1.18)	0.534	1.04 (0.92-1.19)	0.511	1.03 (0.92-1.16)	0.587	1.04 (0.92-1.17)	0.553
**Twin/ multiple birth**								
No	1.00		1.00		1.00		1.00	
Yes	1.74 (0.77-3.93)	0.180	1.75 (0.78-3.90)	0.173	1.28 (0.59-2.75)	0.532	1.28 (0.60-2.74)	0.519
**Age (years)**								
0	1.00		1.00		1.00		1.00	
1	1.18 (0.90-1.54)	0.239	1.18 (0.90-1.55)	0.225	1.07 (0.83-1.38)	0.617	1.07 (0.83-1.38)	0.582
2	0.90 (0.69-1.17)	0.415	0.90 (0.70-1.17)	0.432	0.87 (0.68-1.12)	0.286	0.88 (0.69-1.13)	0.305
3	0.56 (0.42-0.74)	<0.001	0.56 (0.42-0.75)	<0.001	0.58 (0.44-0.77)	<0.001	0.58 (0.44-0.77)	<0.001
4	0.48 (0.34-0.69)	<0.001	0.48 (0.34-0.69)	<0.001	0.50 (0.36-0.71)	<0.001	0.50 (0.36-0.71)	<0.001
**Sex**								
Male	1.00		1.00		1.00		1.00	
Female	0.90 (0.75-1.07)	0.214	0.90 (0.75-1.07)	0.216	0.90 (0.76-1.06)	0.198	0.90 (0.76-1.06)	0.200
**Birth order**								
1	1.00		1.00		1.00		1.00	
2	1.33 (1.07-1.66)	0.011	1.33 (1.07-1.66)	0.010	1.33 (1.07-1.64)	0.009	1.33 (1.07-1.64)	0.009
3	1.94 (1.30-2.90)	0.001	1.95 (1.30-2.90)	0.001	1.88 (1.29-2.76)	0.001	1.89 (1.29-2.76)	0.001
≥4	2.94 (1.58-5.46)	0.001	2.94 (1.58-5.46)	0.001	2.55 (1.41-4.61)	0.002	2.55 (1.41-4.61)	0.002
**Received benefits from *Anganwadi* Centre**								
No	1.00		1.00		1.00		1.00	
Yes	0.93 (0.74-1.16)	0.512	0.93 (0.74-1.16)	0.503	0.95 (0.77-1.18)	0.642	0.95 (0.77-1.17)	0.630
**Received three doses of DPT vaccinations**	nm				nm			
No			1.00				1.00	
Yes			0.94 (0.74-1.21)	0.637			0.93 (0.74-1.18)	0.546
**n (included in the model)**	7,077		7,077		7,395		7,395	
**n (dropped from the model)**	9,599		9,599		9,281		9,281	

nm: not included in the model
BMI: body mass index, CI: confidence interval, DPT: diphtheria pertussis and tetanus, n: sample, OR: odds ratio, p: level of significance

Table S2. Mother fixed-effects regression of anthropometric failure (stunting, wasting, and underweight) on receipt of vitamin A supplementation (VAS) and control variables.

	Stunting				Wasting				Underweight			
	Model I OR (95% CI)	p	Model II OR (95% CI)	p	Model I OR (95% CI)	p	Model II OR (95% CI)	p	Model I OR (95% CI)	p	Model II OR (95% CI)	p
**Received VAS**												
No	1.00		1.00		1.00		1.00		1.00		1.00	
Yes	1.02 (0.91-1.15)	0.694	1.03 (0.92-1.15)	0.663	0.92 (0.80-1.05)	0.226	0.92 (0.80-1.05)	0.231	1.02 (0.92-1.14)	0.685	1.03 (0.92-1.15)	0.609
**Twin/ multiple birth**												
No	1.00		1.00		1.00		1.00		1.00		1.00	
Yes	0.86 (0.28-2.62)	0.792	0.86 (0.28-2.63)	0.796	2.63 (0.76-9.09)	0.125	2.65 (0.77-9.08)	0.120	0.85 (0.37-1.96)	0.703	0.86 (0.37-1.96)	0.713
**Age (years)**												
0	1.00		1.00		1.00		1.00		1.00		1.00	
1	4.55 (3.53-5.85)	<0.001	4.56 (3.54-5.88)	<0.001	0.76 (0.58-1.00)	0.047	0.76 (0.58-1.00)	0.053	2.01 (1.58-2.57)	<0.001	2.03 (1.59-2.60)	<0.001
2	6.67 (5.00-8.90)	<0.001	6.69 (4.99-8.95)	<0.001	0.39 (0.29-0.52)	<0.001	0.39 (0.29-0.53)	<0.001	2.11 (1.63-2.75)	<0.001	2.14 (1.64-2.79)	<0.001
3	7.65 (5.47-10.68)	<0.001	7.66 (5.48-10.71)	<0.001	0.42 (0.30-0.61)	<0.001	0.43 (0.30-0.61)	<0.001	2.49 (1.83-3.40)	<0.001	2.52 (1.85-3.44)	<0.001
4	6.32 (4.24-9.44)	<0.001	6.34 (4.24-9.46)	<0.001	0.34 (0.23-0.52)	<0.001	0.35 (0.23-0.53)	<0.001	2.38 (1.65-3.44)	<0.001	2.40 (1.66-3.46)	<0.001
**Sex**												
Male	1.00		1.00		1.00		1.00		1.00		1.00	
Female	1.02 (0.88-1.18)	0.813	1.02 (0.88-1.18)	0.820	0.86 (0.72-1.03)	0.102	0.86 (0.72-1.03)	0.103	0.96 (0.83-1.12)	0.633	0.96 (0.83-1.12)	0.611
**Birth order**												
1	1.00		1.00		1.00		1.00		1.00		1.00	
2	2.19 (1.72-2.79)	<0.001	2.19 (1.72-2.78)	<0.001	0.89 (0.69-1.16)	0.398	0.89 (0.69-1.16)	0.398	1.33 (1.06-1.68)	0.014	1.33 (1.06-1.68)	0.015
3	3.99 (2.57-6.21)	<0.001	3.99 (2.57-6.20)	<0.001	1.10 (0.68-1.77)	0.700	1.10 (0.68-1.77)	0.702	2.09 (1.37-3.18)	0.001	2.07 (1.36-3.17)	0.001
≥4	8.53 (4.24-17.17)	<0.001	8.53 (4.24-17.16)	<0.001	1.13 (0.55-2.34)	0.732	1.13 (0.55-2.34)	0.737	3.29 (1.71-6.33)	<0.001	3.28 (1.70-6.32)	<0.001
**Received benefits from *Anganwadi* Centre**												
No	1.00		1.00		1.00		1.00		1.00		1.00	
Yes	0.76 (0.61-0.96)	0.019	0.76 (0.61-0.96)	0.019	1.23 (0.96-1.57)	0.097	1.23 (0.96-1.58)	0.095	0.93 (0.75-1.16)	0.541	0.94 (0.75-1.16)	0.556

**Received three doses of DPT vaccinations**	nm			nm			nm		
No		1.00			1.00			1.00	
Yes		0.97 (0.78-1.22)	0.813		0.97 (0.73-1.28)	0.807		0.90 (0.71-1.13)	0.345
**n (included in the model)**	6,619	6,619		4,345	4,345		5,844	5,844	
**n (dropped from the model)**	9,679	9,679		11,953	11,953		10,454	10,454	

nm: not included in the model

BMI: body mass index, CI: confidence interval, DPT: diphtheria pertussis and tetanus, n: sample, OR: odds ratio, p: level of significance

Table S3. Sample distribution (sample included to estimate household fixed-effects) of anaemia status among children who received vitamin A versus children who did not receive vitamin A by select characteristics of children.

	**Presence of any anaemia**		**Absence of any anaemia**		**Presence of moderate/mild anaemia**		**Absence of moderate/mild anaemia**	
	vitamin A received	vitamin A not received	vitamin A received	vitamin A not received	vitamin A received	vitamin A not received	vitamin A received	vitamin A not received
**Twin/ multiple birth**								
No	98.1	98.6	98.6	99.1	98.2	98.6	98.5	98.8
Yes	1.9	1.4	1.4	0.9	1.9	1.4	1.5	1.2
**Age (years)**								
0	14.6	27.9	9.3	12.7	14.8	27.2	9.5	12.9
1	38.8	22.6	18.8	8.8	37.9	21.8	20.6	9.6
2	24.2	16.6	19.6	13.1	23.9	16.4	19.7	13.7
3	12.6	15.9	25.7	26.8	13.2	16.7	24.8	26.6
4	9.8	17.1	26.6	38.6	10.3	17.9	25.5	37.3
**Sex**								
Male	51.1	51.5	47.0	48.1	50.6	51.5	47.7	48.2
Female	48.9	48.5	53.0	51.9	49.4	48.5	52.3	51.8
**Birth order**								
1	21.4	27.5	37.9	43.4	21.7	28.4	36.8	42.7
2	41.6	37.6	33.3	31.9	41.6	36.8	33.8	31.9
3	21.6	18.5	15.8	13.9	21.4	18.3	15.8	14.2
≥4	15.4	16.4	13.0	10.7	15.3	16.5	13.6	11.2
**Received benefits from *Anganwadi* Centre**								
No	36.6	45.8	41.0	48.0	37.1	45.9	41.0	48.1
Yes	63.4	54.2	59.0	51.96	62.9	54.1	59.0	51.9
**n**	**2,785**	**2,359**	**2,249**	**2,629**	**2,874**	**2,489**	**2,386**	**2,715**

n: sample

Table S4. Sample distribution (sample included to estimate household fixed-effects) of anthropometric failure among children who received vitamin A versus children who did not receive vitamin A by select characteristics of children.

	Stunted		Not stunted		Wasted		Not wasted		Underweight		Not underweight	
	vitamin A received	vitamin A not received	vitamin A received	vitamin A not received	vitamin A received	vitamin A not received	vitamin A received	vitamin A not received	vitamin A received	vitamin A not received	vitamin A received	vitamin A not received
**Twin/ multiple birth**												
No	98.5	99.1	98.3	98.9	98.3	98.5	98.4	99.3	98.0	98.4	97.6	99.0
Yes	1.5	0.9	1.7	1.1	1.7	1.5	1.6	0.8	2.0	1.6	2.4	1.0
**Age (years)**												
0	6.2	11.7	19.1	30.3	19.9	33.5	8.4	14.5	10.0	17.2	14.7	24.1
1	29.5	18.4	27.9	12.4	35.3	19.5	23.4	11.6	29.2	16.6	28.1	13.7
2	26.8	20.1	17.5	12.4	18.6	12.7	24.0	19.0	22.3	18.0	21.1	14.6
3	22.4	26.8	17.6	17.6	14.8	17.7	24.2	26.1	22.0	23.6	18.4	21.4
4	15.0	23.0	17.9	27.4	11.5	16.7	20.0	28.8	16.6	24.6	17.6	26.2
**Sex**												
Male	50.1	48.4	48.4	51.3	56.8	51.6	49.2	47.9	50.9	46.6	49.5	47.9
Female	49.9	51.6	51.6	48.7	43.2	48.4	50.9	52.1	49.1	53.4	50.5	52.1
**Birth order**												
1	29.7	33.6	29.8	33.3	24.1	29.1	35.6	39.2	29.3	34.8	32.2	32.6
2	35.9	34.1	36.2	35.5	37.8	37.2	33.7	32.8	34.2	32.8	35.9	37.1
3	17.7	16.4	20.2	17.6	20.2	19.2	17.1	13.9	18.6	17.3	19.0	15.8
≥4	16.7	15.9	13.8	13.7	17.9	14.5	13.7	14.1	17.9	15.1	13.0	14.5
**Received benefits from *Anganwadi* Centre**												
No	36.1	47.3	36.2	42.4	29.1	41.0	35.8	43.2	33.3	45.7	36.4	42.4
Yes	63.9	52.8	63.8	57.6	70.9	59.0	64.2	56.9	66.7	54.3	63.6	57.6
**n**	**2,490**	**2,161**	**2,236**	**2,506**	**1,429**	**1,382**	**1,662**	**1,714**	**2,156**	**2,025**	**2,118**	**2,201**

n: sample
